# Supplementary material for: ALDH7A1 inhibits the intracellular transport pathways during hypoxia and starvation to promote cellular energy homeostasis
Source: Nat Commun. 2019 Sep 6;10:4068. doi: 10.1038/s41467-019-11932-0 (PMC6731274; doi:10.1038/s41467-019-11932-0)
Supplement: Supplementary file 1 — Supplementary Information [file 41467_2019_11932_MOESM1_ESM.pdf]

SUPPLEMENTARY INFORMATION

ALDH7A1 inhibits the intracellular transport pathways during hypoxia and starvation to promote cellular energy homeostasis

(Yang et al)

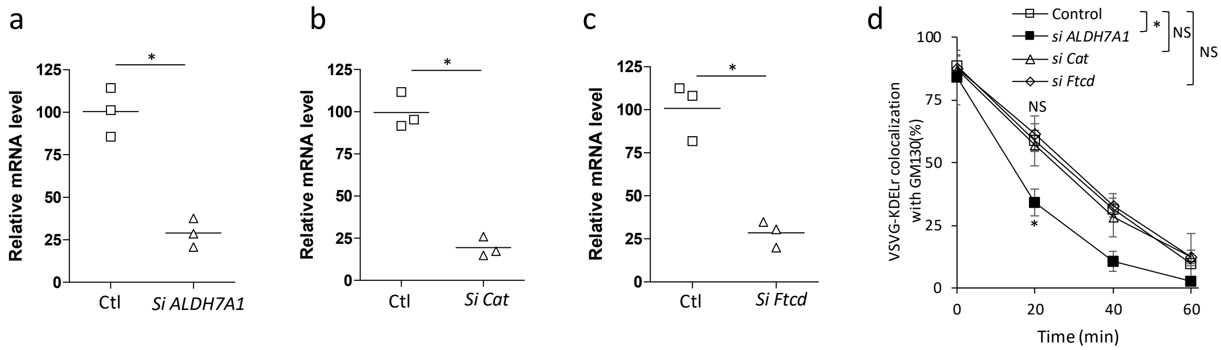

Supplementary Figure 1. Assessing the role of metabolic enzymes in COPI transport. Source data are provided as a Source Data file. **(a)** Efficiency of siRNA against *ALDH7A1* in HeLa cells was assessed by quantitative polymerase chain reaction (qPCR); n = 3 independent experiments. **(b)** Efficiency of siRNA against *catalase (Cat)* in HeLa cells was assessed by qPCR; n = 3 independent experiments. **(c)** Efficiency of siRNA against *Ftcd* was assessed by qPCR; n = 3 independent experiments. **(d)** HeLa cells were treated as indicated, and then colocalization of VSVG-KDELR with a cis-Golgi marker (GM130) coupled with kinetic analysis was performed. Mean with standard deviation; * $p<0.05$, NS (not significant), paired two-tailed Student's t-test; n = 3 independent experiments.

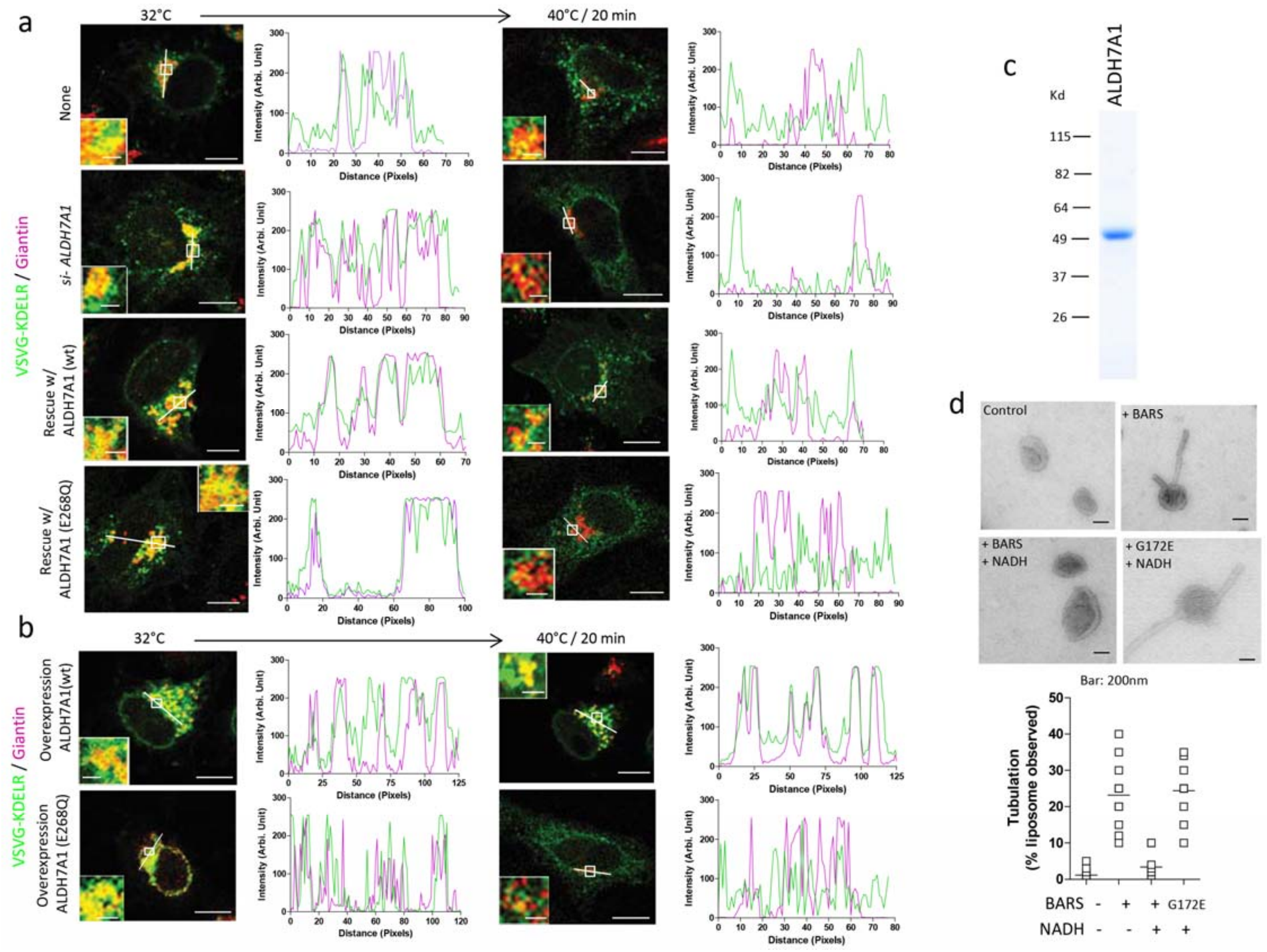

Supplementary Figure 2. Further characterizing the inhibition of COPI transport by ALDH7A1. Source data are provided as a Source Data file. **(a)** Representative confocal images for data shown in Fig 1d, VSVG-KDELR (green) and giantin (red), bar = 10 um (2 um in inset). Quantitative colocalization along a line plot is also shown. **(b)** Representative confocal images for data shown in Fig 1e, VSVG-KDELR (green) and giantin (red), bar = 10 um (2 um in inset). Quantitative colocalization along a line plot is also shown. **(c)** Purity of recombinant of ALDH7A1 as assessed by SDS/PAGE followed by coomassie blue staining, n = 2 independent experiments with a representative result shown. **(d)** Liposomes were subjected to different conditions as indicated, followed by EM examination. Representative images are shown above (bar = 200 nm), and quantitation is shown below. Mean with standard deviation is shown; * $p<0.05$, paired two-tailed Student's t-test; n = 3 independent experiments.

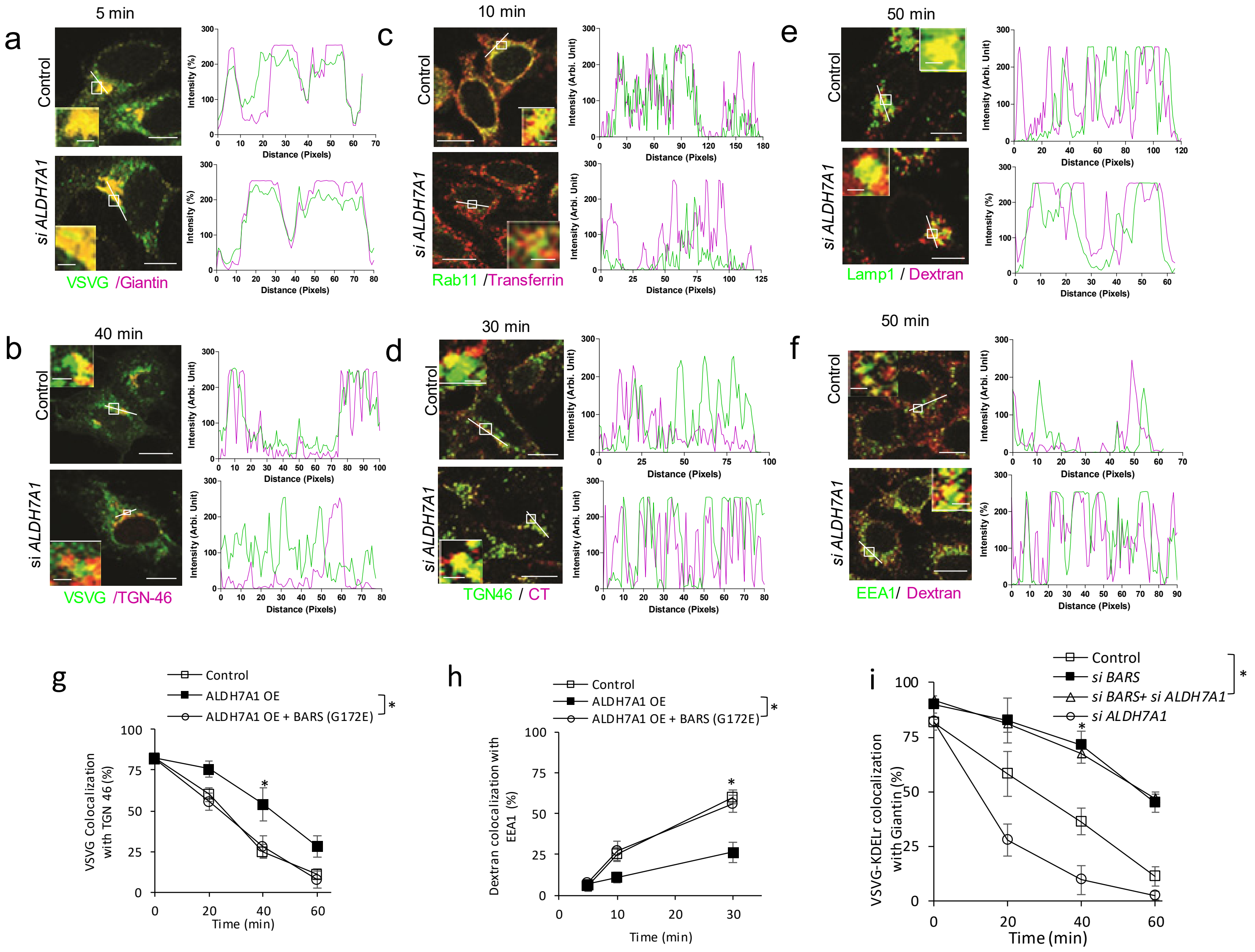

Supplementary Figure 3. Further characterizing the inhibition of transport pathway by ALDH7A1. Quantitative data are shown as mean with standard deviation; * p<0.05, paired two-tailed Student's t-test. Source data are provided as a Source Data file. **(a)** Representative confocal images for the data shown in Fig 2a, VSVG (green) and giantin (red), bar = 10 um (2 um in inset). A line plot is also shown. **(b)** Representative confocal images for the data shown in Fig 2b, VSVG (green) and TGN46 (red), bar = 10 um (2 um in inset). Quantitative colocalization along a line plot is also shown as example. **(c)** Representative confocal images for the data shown in Fig 2c, Rab11 (green) and transferrin (red), bar = 10 um (2 um in inset). A line plot is also shown. **(d)** Representative confocal images for the data shown in Fig 2d, TGN46 (green) and CT (red), bar = 10 um (2 um in inset). A line plot is also shown. **(e)** Representative confocal images for the data shown in Fig 2e, Lamp1 (green) and dextran (red), bar = 10 um (2 um in inset). A line plot is also shown. **(f)** Representative confocal images for the data shown in Fig 2f, EEA1 (green) and dextran (red), bar = 10 um (2 um in inset). A line plot is also shown. **(g)** HeLa cells were treated as indicated, and then transport from the Golgi to the plasma membrane was assessed; n = 3 independent experiments **(h)** HeLa cells were treated as indicated, and then fluid-phase uptake was assessed; n = 3 independent experiments. **(i)** HeLa cells were treated as indicated, and then COPI transport from the Golgi to the ER was assessed; n = 3 independent experiments.

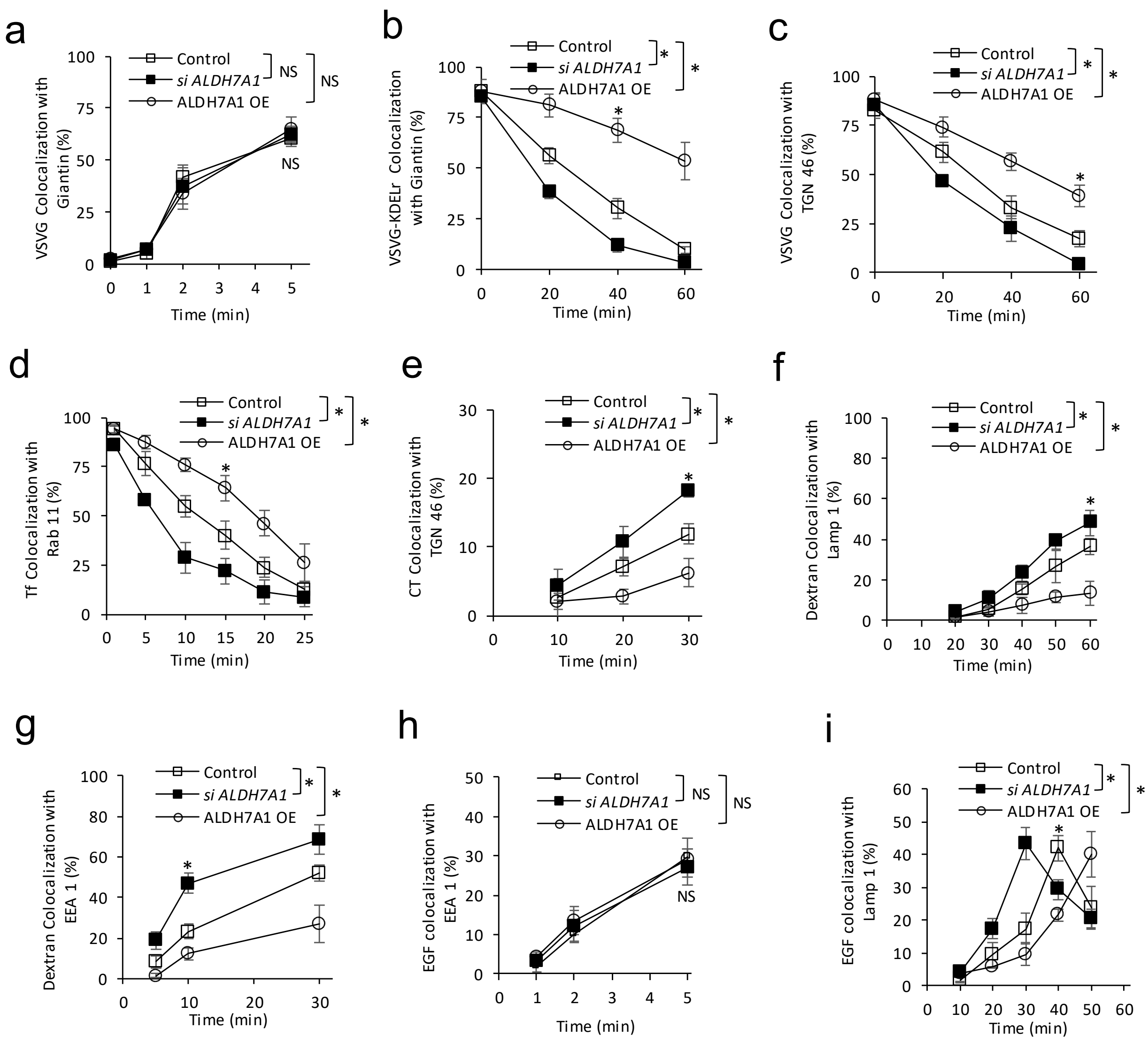

Supplementary Figure 4. ALDH7A1 inhibits the identical transport pathways in HEK293 cells. Quantitative results are shown as mean with standard deviation; * $p<0.05$, NS (not significant), paired two-tailed Student's t-test; n = 3 independent experiments. Source data are provided as a Source Data file. HEK293 cells were assessed for the following transport pathways: **(a)** Transport of VSVG from the ER to the Golgi was assessed through the quantitative colocalization of VSVG with a cis-Golgi marker (giantin). **(b)** COPI transport from the Golgi to the ER was assessed through the quantitative colocalization of VSVG-KDELR with a cis-Golgi marker (giantin). **(c)** Transport of VSVG from the Golgi to the plasma membrane was assessed through the quantitative colocalization of VSVG with a TGN marker (TGN46). **(d)** Endocytic recycling of Tf from the recycling endosome (RE) to the plasma membrane was assessed through the quantitative colocalization of internalized Tf with a recycling endosome marker (Rab11). **(e)** Endocytic transport of CT to the Golgi was assessed through the quantitative colocalization of internalized CT with a TGN marker (TGN46). **(f)** Endocytic transport of internalized dextran to the lysosome was assessed through the quantitative colocalization of internalized dextran with a lysosome marker (Lamp1). **(g)** Fluid-phase endocytosis of dextran was assessed through the quantitative colocalization of internalized dextran with an early endosome marker (EEA1). **(h)** Endocytosis of EGFR was assessed through the quantitative colocalization of internalized EGF with an early endosome marker (EEA1). **(i)** Endocytic transport of internalized EGFR to the lysosome was assessed through the quantitative colocalization of internalized EGF with a lysosome marker (Lamp1).

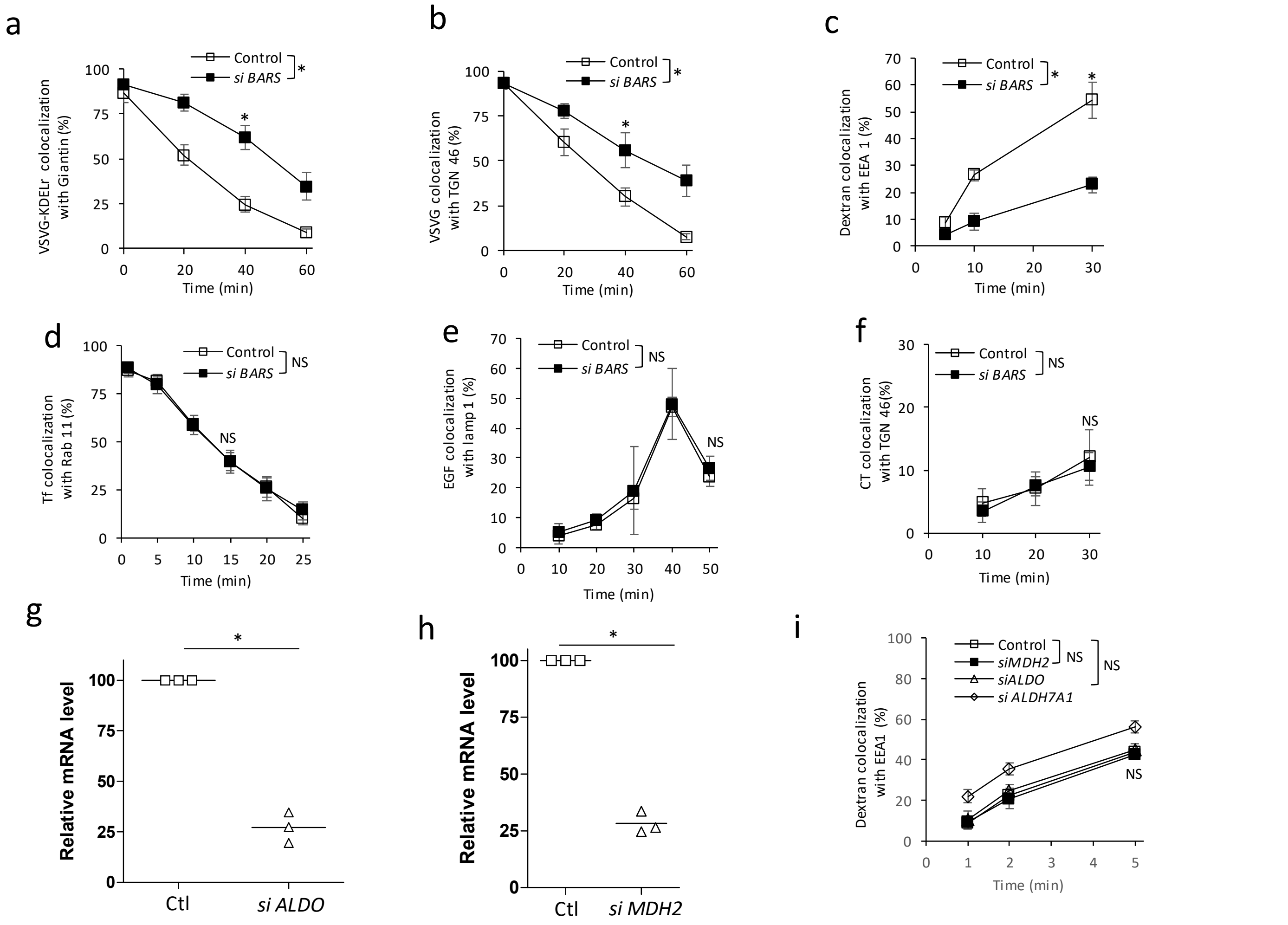

Supplementary Figure 5. Examining transport pathways regulated by BARS and metabolic enzymes. Quantitative results are shown as mean with standard deviation; * $p<0.05$, NS (not significant), paired two-tailed Student's t-test; n = 3 independent experiments. Source data are provided as a Source Data file. **(a)** COPI transport from the Golgi to the ER in HeLa cells was assessed through the quantitative colocalization of VSVG-KDELR with a cis-Golgi marker (giantin). **(b)** Transport of VSVG from the Golgi to the plasma membrane in HeLa cells was assessed through the quantitative colocalization of VSVG with a TGN marker (TGN46). **(c)** Fluid-phase endocytosis of dextran in HeLa cells was assessed through the quantitative colocalization of internalized dextran with an early endosome marker (EEA1). (d) Endocytic recycling of Tf from the recycling endosome (RE) to the plasma membrane in HeLa cells was assessed through the quantitative colocalization of internalized Tf with a recycling endosome marker (Rab11). **(e)** Endocytic transport of internalized EGFR to the lysosome in HeLa cells was assessed through the quantitative colocalization of internalized EGF with a lysosome marker (Lamp1). **(f)** Endocytic transport of CT to the Golgi in HeLa cells was assessed through the quantitative colocalization of internalized CT with a TGN marker (TGN46). **(g)** Efficiency of siRNA against *aldolase* (ALDO) in HeLa cells as assessed by qPCR. **(h)** Efficiency of siRNA against *malate dehydrogenase 2* (MDH2) in HeLa cells as assessed by qPCR. **(i)** HeLa cells were treated as indicated, and then colocalization of dextran with an early endosome marker (EEA1) coupled with kinetic analysis was performed.

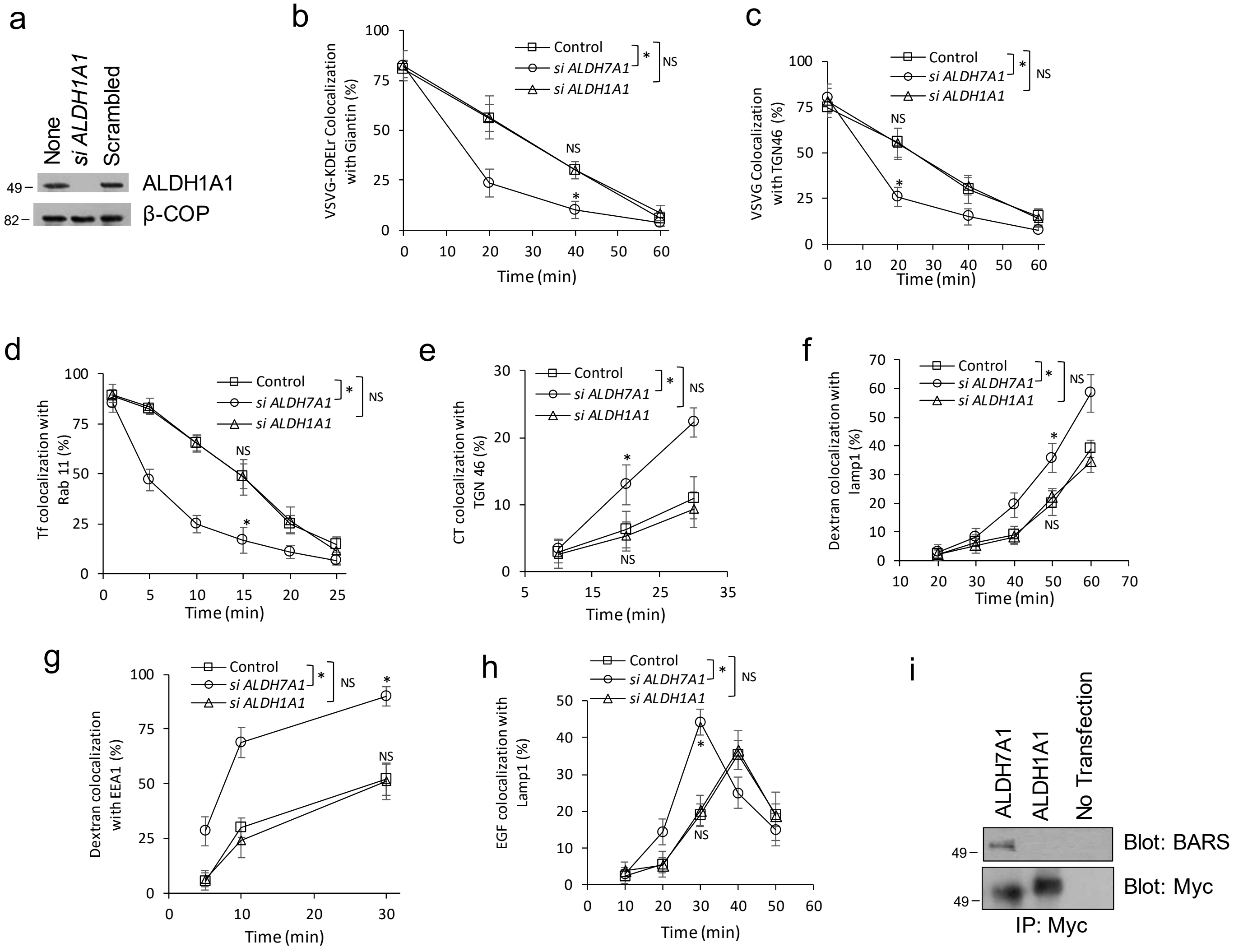

Supplementary Figure 6. ALDH1A1 does not regulate the intracellular pathways. Quantitative results are shown as mean with standard deviation; * $p<0.05$, NS (not significant), paired two-tailed Student's t-test; n = 3 independent experiments. Source data are provided as a Source Data file. **(a)** HeLa cells were treated as indicated, followed by immunoblotting for proteins as indicated; n = 2 experiments with a representative result shown. **(b)** COPI transport in HeLa cells was assessed through the quantitative colocalization of VSVG-KDELR with a cis-Golgi marker (giantin). **(c)** Transport from the Golgi to the plasma membrane in HeLa cells was assessed through the quantitative colocalization of VSVG with a TGN marker (TGN46). **(d)** Endocytic recycling of TfR in HeLa cells was assessed through the quantitative colocalization of internalized Tf with a recycling endosome marker (Rab11). **(e)** Endocytic transport of CT to the Golgi in HeLa cells was assessed through the quantitative colocalization of internalized CT with a TGN marker (TGN46). **(f)** Endocytic transport of dextran to the lysosome in HeLa cells was assessed through the quantitative colocalization of internalized dextran with a lysosome marker (Lamp1). **(g)** Fluid-phase uptake in HeLa cells was assessed through the quantitative colocalization of internalized dextran with an early endosome marker (EEA1). **(h)** Endocytic transport of EGFR to the lysosome in HeLa cells was assessed through the quantitative colocalization of internalized EGF with a lysosome marker (Lamp1). **(i)** ALDH1A1 does not interact with BARS in cells. HeLa cells were transfected with myc-tagged constructs as indicated, followed by immunoprecipitation for these constructs and then immunoblotting for proteins as indicated; n = 2 experiments with a representative result shown.

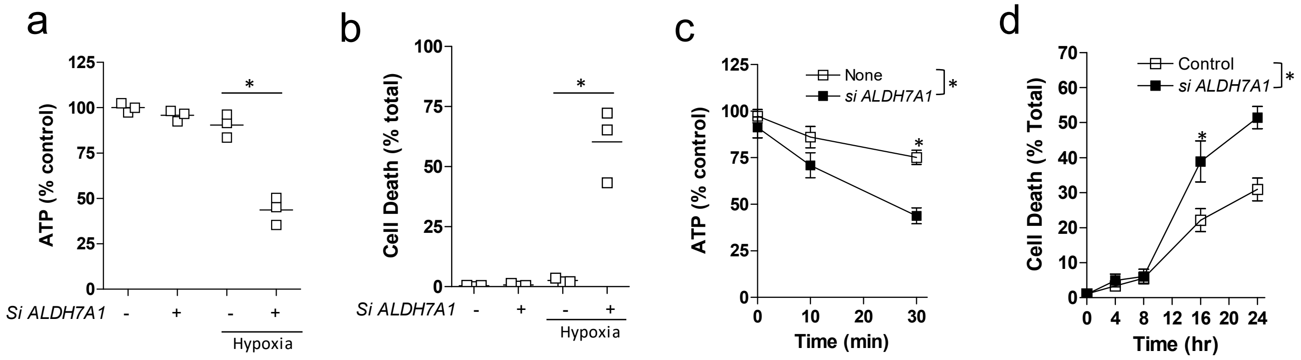

Supplementary Figure 7. ALDH7A1 promotes energy homeostasis in HEK293 cells. Quantitative results are shown as mean with standard deviation; * $p<0.05$, two-tailed Student's t-test; n = 3 independent experiments. Source data are provided as a Source Data file. **(a)** Cells were treated as indicated, and then total ATP level was quantified. **(b)** Cells were treated as indicated, and then cell death was quantified. **(c)** Cells were treated with starvation conditions as indicated, and then total ATP level was quantified. **(d)** Cells were treated with starvation conditions as indicated, and then cell death was quantified.

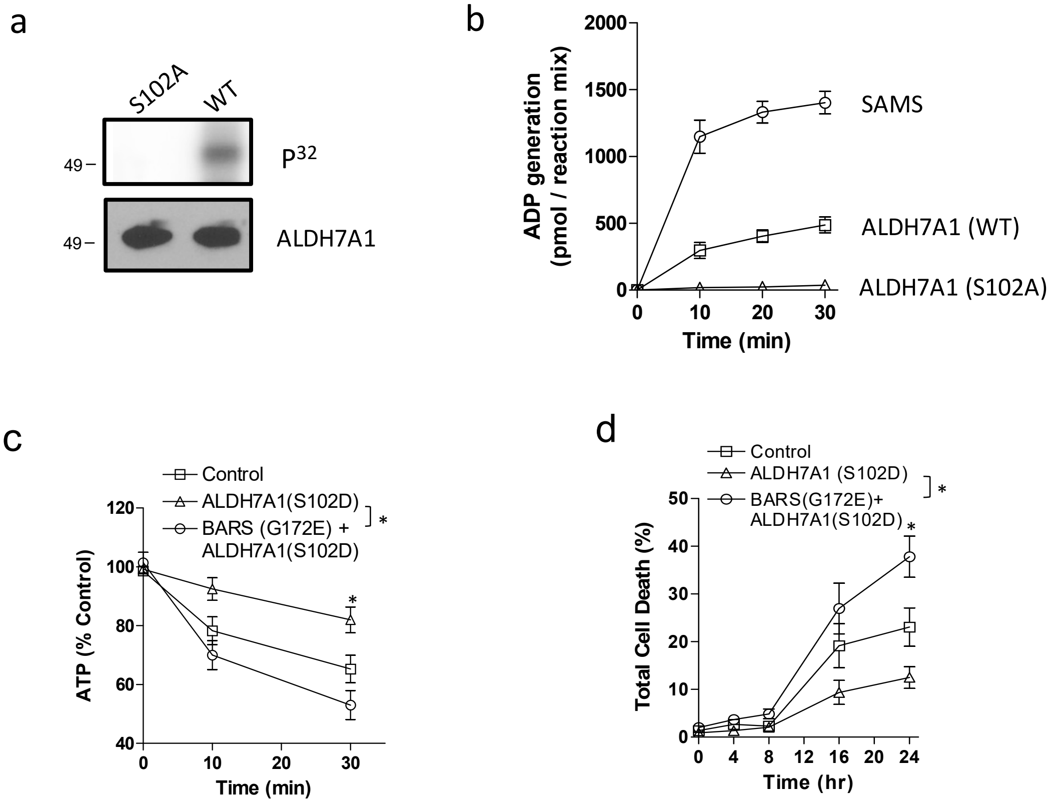

Supplementary Figure 8. Further characterizing the phosphorylation of ALDH7A1 and its effects. Source data are provided as a Source Data file. **(a)** The in vitro kinase assay was performed using recombinant forms of AMPK and ALDH7A1 in the presence of γ-labeled P^{32}-ATP; n = 2 independent experiments with a representative result shown. **(b)** The in vitro kinase assay was performed using recombinant forms of AMPK and ALDH7A1, followed by quantitation of ADP generated by the reaction; n = 3 independent experiments. The SAMS peptide, a sequence from an optimal AMPK substrate, Acetyl-CoA Carboxylase (ACC), was used for comparison. **(c)** HeLa cells were treated with starvation conditions as indicated, followed by quantitation of total ATP level. Mean with standard deviation is shown; n = 3 independent experiments. **(d)** HeLa cells were treated with starvation conditions as indicated, followed by quantitation of cell death. Mean with standard deviation is shown; n = 3 independent experiments.

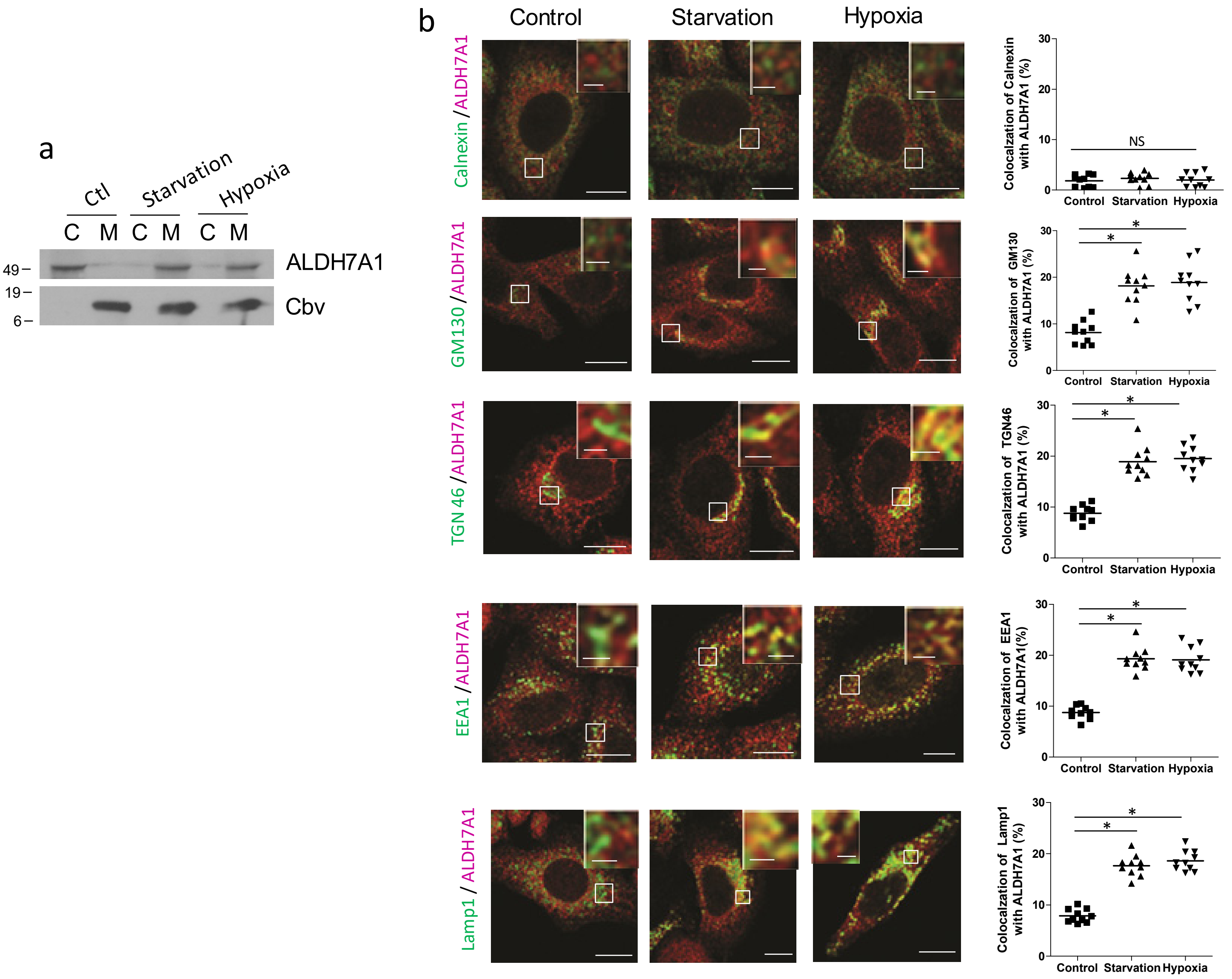

Supplementary Figure 9. Redistribution of ALDH7A1 by hypoxia and starvation. (a) HeLa cells were treated as indicated. The cytoplasmic fraction was then subjected to centrifugation to obtain total membranes (M) and cytosol (C), followed by immunoblotting for proteins as indicated. Cellubrevin (Cbv) serves as a marker for membranes. A representative result from 2 independent experiments is shown. **(b)** Confocal microscopy was performed on HeLa cells to detect the colocalization of ALDH7A1 with different organelle markers. Representative images are shown (left); bar, 10 um (2 um in inset). Colocalization results are also quantified (right); mean with standard deviation, * $p<0.05$, NS (not significant), paired two-tailed Student's t-test; n = 2 independent experiments with a representative experiment shown.

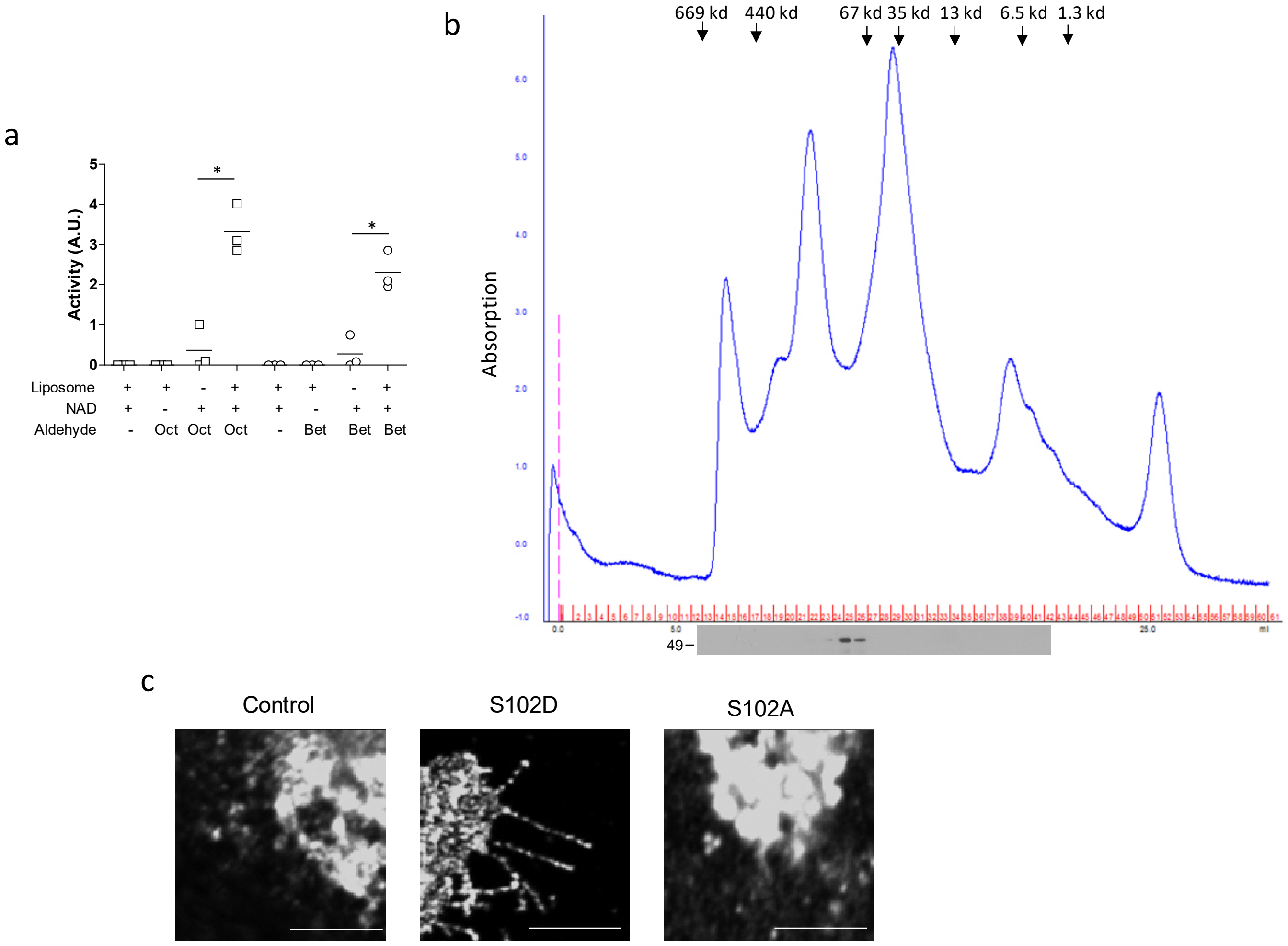

Supplementary Figure 10. Further characterizing the effects of ALDH7A1 activity. Source data are provided as a Source Data file. **(a)** Liposomes were generated using purified lipids to mimic the composition of Golgi membrane. Substrate (either octanal or betaine aldehyde) and NAD were then added, followed by incubation with recombinant ALDH7A1. The level of NADH generated was then quantified. Mean with standard deviation is shown; * $p<0.05$, paired two-tailed Student's t-test; $n = 3$ independent experiments. **(b)** Golgi membrane was incubated with the S102D mutant of ALDH7A1, followed by centrifugation to obtain membrane versus soluble fractions. The soluble fraction was then subjected to gel filtration, and BARS was tracked by immunoblotting of eluted fractions. When compared to marker proteins, BARS eluted as dimers. A representative result from 2 independent experiments is shown. **(c)** HeLa cells that expressed the different mutant forms of ALDH7A1 as indicated, which was achieved by siRNA against *ALDH7A1* followed by transfection of mutant forms, were examined by immunofluorescence microscopy that tracked VSVG leaving the TGN; $n = 3$ independent experiments with a representative result shown.

Supplementary Table 1. Proteins that interact with BARS. Highlighted in red are metabolic enzymes.

Abbreviation	MWT(kDa)	Protein Name
Eprs	166.68	LRRGT00050 OS=Rattus norvegicus GN=Eprs PE=1 SV=1
Copa	138.27	Coatomer subunit alpha OS=Rattus norvegicus GN=Copa PE=1 SV=1
Ap2a2	103.98	AP-2 complex subunit alpha-2 OS=Rattus norvegicus GN=Ap2a2 PE=1 SV=3
Copb2	102.49	Coatomer subunit beta' OS=Rattus norvegicus GN=Copb2 PE=1 SV=3
Copg1	97.55	Coatomer subunit gamma-1 OS=Rattus norvegicus GN=Copg1 PE=2 SV=1
Sec23a	86.11	Protein Sec23a OS=Rattus norvegicus GN=Sec23a PE=1 SV=1
Cat	59.72	Catalase OS=Rattus norvegicus GN=Cat PE=1 SV=3
Cct7	59.62	Protein Cct7 OS=Rattus norvegicus GN=Cct7 PE=1 SV=1
Cct8	59.55	Chaperonin subunit 8 (Theta) (Predicted), isoform CRA_a OS=Rattus norvegicus GN=Cct8 PE=1 SV=1
Ftcd	58.88	Formimidoyltransferase-cyclodeaminase OS=Rattus norvegicus GN=Ftcd PE=1 SV=4
Aldh7a1	58.71	Alpha-aminoadipic semialdehyde dehydrogenase OS=Rattus norvegicus GN=Aldh7a1 PE=1 SV=2
Rab11b	24.47	Ras-related protein Rab-11B OS=Rattus norvegicus GN=Rab11b PE=1 SV=4
Sar1b	22.4	GTP-binding protein SAR1b OS=Rattus norvegicus GN=Sar1b PE=2 SV=1

Supplementary Table 2. Stoichiometry of phosphorylation.

	Stoichiometry of maximal phosphorylation (mol phosphorylation / mol total substrate)
SAMS<br>ALDH7A1	0.801 ± 0.080<br>0.566 ± 0.092

Supplementary Table 3. ALDH7A1 activity, Km

	Octanal	Betaine aldehyde
No Liposome	24.89±1.30	52.2±1.50
With Liposome	0.25±0.08	0.55±0.06
