# Supplementary material for: ALDH7A1 inhibits the intracellular transport pathways during hypoxia and starvation to promote cellular energy homeostasis
Source: Nat Commun. 2019 Sep 6;10:4068. doi: 10.1038/s41467-019-11932-0 (PMC6731274; doi:10.1038/s41467-019-11932-0)
Supplement: Supplementary file 3 — Reporting Summary [file 41467_2019_11932_MOESM3_ESM.pdf]

nature research

Corresponding author(s): Victor W. Hsu

Last updated by author(s): Jun 10, 2019

Reporting Summary

Nature Research wishes to improve the reproducibility of the work that we publish. This form provides structure for consistency and transparency in reporting. For further information on Nature Research policies, see Authors & Referees and the Editorial Policy Checklist.

Statistics

For all statistical analyses, confirm that the following items are present in the figure legend, table legend, main text, or Methods section.

n/a	Confirmed	
☐	☒	The exact sample size (*n*) for each experimental group/condition, given as a discrete number and unit of measurement
☐	☒	A statement on whether measurements were taken from distinct samples or whether the same sample was measured repeatedly
☐	☒	The statistical test(s) used AND whether they are one- or two-sided<br>*Only common tests should be described solely by name; describe more complex techniques in the Methods section.*
☒	☐	A description of all covariates tested
☐	☒	A description of any assumptions or corrections, such as tests of normality and adjustment for multiple comparisons
☐	☒	A full description of the statistical parameters including central tendency (e.g. means) or other basic estimates (e.g. regression coefficient) AND variation (e.g. standard deviation) or associated estimates of uncertainty (e.g. confidence intervals)
☐	☒	For null hypothesis testing, the test statistic (e.g. *F*, *t*, *r*) with confidence intervals, effect sizes, degrees of freedom and *P* value noted<br>*Give P values as exact values whenever suitable.*
☒	☐	For Bayesian analysis, information on the choice of priors and Markov chain Monte Carlo settings
☒	☐	For hierarchical and complex designs, identification of the appropriate level for tests and full reporting of outcomes
☒	☐	Estimates of effect sizes (e.g. Cohen's *d*, Pearson's *r*), indicating how they were calculated

Our web collection on statistics for biologists contains articles on many of the points above.

Software and code

Policy information about availability of computer code

Data collection	Colocalization was performed using the Nikon EZ-C1 version 3.90 acquisition software or the Zeiss Zen 2.3 blue edition confocal acquisition software.
Data analysis	Quantitation of colocalization used Image J. Statistical analysis used Prism or Excel.

For manuscripts utilizing custom algorithms or software that are central to the research but not yet described in published literature, software must be made available to editors/reviewers. We strongly encourage code deposition in a community repository (e.g. GitHub). See the Nature Research guidelines for submitting code & software for further information.

Data

Policy information about availability of data

All manuscripts must include a data availability statement. This statement should provide the following information, where applicable:

- Accession codes, unique identifiers, or web links for publicly available datasets
- A list of figures that have associated raw data
- A description of any restrictions on data availability

The data that support the findings of this study are available from the corresponding author upon reasonable request.

Field-specific reporting

Please select the one below that is the best fit for your research. If you are not sure, read the appropriate sections before making your selection.

☒ Life sciences ☐ Behavioural & social sciences ☐ Ecological, evolutionary & environmental sciences

Life sciences study design

All studies must disclose on these points even when the disclosure is negative.

Sample size	Sample size was based on our previous experience in the experimental approach, and also taking into account feasibility, in obtaining reliable results.
Data exclusions	No data were excluded.
Replication	Findings were reliably reproduced.
Randomization	The experimental approaches did not require samples to be randomized. Samples, and the subsequent data collection and analysis, were handled the same way in all experiments.
Blinding	The investigators were not blinded to group allocation during data collection or subsequent analysis. This approach is considered standard for biochemical and microscopy experiments done in this study.

Reporting for specific materials, systems and methods

We require information from authors about some types of materials, experimental systems and methods used in many studies. Here, indicate whether each material, system or method listed is relevant to your study. If you are not sure if a list item applies to your research, read the appropriate section before selecting a response.

Materials & experimental systems

n/a	Involved in the study	
☐	☒	Antibodies
☐	☒	Eukaryotic cell lines
☒	☐	Palaeontology
☒	☐	Animals and other organisms
☒	☐	Human research participants
☒	☐	Clinical data

Methods

n/a	Involved in the study	
☒	☐	ChIP-seq
☒	☐	Flow cytometry
☒	☐	MRI-based neuroimaging

Antibodies

Antibodies used	Rabbit antibody against human ALDH7A1 (WB: 1:1000, IF: 1:500) was generated by injecting the recombinant protein into rabbits using a commercial service (Proteintech). The following antibodies have been described in our previous studies: ARF1 (WB 1:1000), BARS (WB 1:1000), coatomer (IF 1:10), beta-COP (WB 1:10), calnexin (IF 1:200), cellubrevin (WB 1:1000), giantin (IF 1:2000), GM130 (IF 1:500), Lamp1 (IF 1:200), Sec61p (IF 1:500), TGN46 (IF 1:200), VSVG (IF 1:10), HA epitope tag (WB 1:200), and Myc epitope tag (WB 1:200). The following antibodies were obtained from commercial sources: actin (WB 1:1000, Ambion, AM4302), EEA1 (1:100 IF, BD Transduction Laboratories, 610456), GFP (WB 1:1000, Thermo Scientific, MA5-15256), GST (WB 1:1000, Santa Cruz Biotechnology, sc-138), and Rab11 (IF 1:200, BD Biosciences, 610656). Other primary antibodies were obtained from Cell Signaling: ALDH1A1 (12035S, WB 1:1000), AMPK-alpha (2603S, WB 1:500), phospho-substrate-AMPK antibody (5759S, WB 1:500), Lamin B (12586S, WB 1:1000), and Sirt1 (2493S, WB 1:500). Conjugated secondary antibodies were obtained from Jackson ImmunoResearch: horseradish peroxidase-conjugated donkey antibodies against mouse IgG (715-035-150, WB 1:10,000) and against rabbit IgG (711-035-152, WB 1:10,000), Cy2 donkey antibodies against mouse IgG (715-225-151, IF 1:200) and against rabbit IgG (711-225-152, IF 1:200), Cy3 goat antibody against mouse IgG (115-165-062, IF 1:200), Cy3 donkey antibodies against rabbit IgG (711-165-152, IF 1:200) and against sheep IgG (713-165-147, IF 1:200).
Validation	For antibodies that have been validated in our previous studies, see Bai, M. et al. Nature Cell Biol 13, 559-567 (2011), Dai, J. et al. Dev Cell 7, 771-776 (2004), Yang, J.S. et al. J Cell Biol 159, 69-78 (2002), Yang, J.S. et al. EMBO J 24, 4133-4143 (2005). For antibodies obtained from commercial sources listed in the above section, validation was performed by the vendors. Antibody generated against ALDH7A1 was validated in vitro by its recognition of recombinant ALDH7A1 and in cells by the loss of recognition upon siRNA against ALDH7A1.

Eukaryotic cell lines

Policy information about cell lines

Cell line source(s)	HeLa and HEK293 were obtained from ATCC.
Authentication	Cells were authenticated by ATCC.
Mycoplasma contamination	Cells were documented by ATCC to be free of mycoplasma contamination.

Commonly misidentified lines<br>(See ICLAC register)	No commonly misidentified cell lines were used.
